# Transcriptional regulation of macrophages in heart failure

**DOI:** 10.3389/fcvm.2023.1148041

**Published:** 2023-03-30

**Authors:** Keyan Wang, Xiaoqian Sun, Ying Sun, Boyang Jiao, Junkai Yao, Yueyao Hu, Qiong Deng, Jianteng Dong, Wei Wang, Yong Wang, Chun Li

**Affiliations:** ^1^ College of Chinese Medicine, Beijing University of Chinese Medicine, Beijing, China; ^2^ Beijing Key Laboratory of Traditional Chinese Medicine (TCM) Syndrome and Formula, Beijing University of Chinese Medicine, Beijing, China; ^3^ School of Chinese Materia Medica, Guangzhou University of Chinese Medicine, Guangzhou, China; ^4^ School of Life Sciences, Beijing University of Chinese Medicine, Beijing, China; ^5^ Modern Research Center for Traditional Chinese Medicine (TCM), Beijing University of Chinese Medicine, Beijing, China

**Keywords:** macrophages, DNA methylation, histone modification, transcription factors, myocardial infarction, adverse cardiac remodeling

## Abstract

Adverse cardiac remodeling after acute myocardial infarction is the most important pathological mechanism of heart failure and remains a major problem in clinical practice. Cardiac macrophages, derived from tissue resident macrophages and circulating monocyte, undergo significant phenotypic and functional changes following cardiac injury and play crucial roles in inflammatory response and tissue repair response. Currently, numerous studies indicate that epigenetic regulatory factors and transcription factors can regulate the transcription of inflammatory and reparative genes and timely conversion of inflammatory macrophages into reparative macrophages and then alleviate cardiac remodeling. Accordingly, targeting transcriptional regulation of macrophages may be a promising option for heart failure treatment. In this review, we not only summarize the origin and function of cardiac macrophages, but more importantly, describe the transcriptional regulation of macrophages in heart failure, aiming to provide a potential therapeutic target for heart failure.

## 1. Introduction

Acute myocardial infarction (AMI) has always been a disease with high morbidity and mortality worldwide. Although reperfusion therapy is currently the main therapeutic strategy, the risk of progression to adverse cardiac remodeling and subsequently heart failure (HF) remains (1, 2). Therefore, it is necessary to explore novel therapeutic targets to effectively alleviate cardiac remodeling in order to protect the heart from failure.

Studies revealed convincing results to the effect that immunotherapy represented by macrophages has significant implications for the treatment of ischemic heart disease (3). Following AMI, dead cardiomyocytes release endogenous damage-associated molecular patterns (DAMPs), which interact with toll-like receptors to activate inflammatory signaling pathways, produce large amounts of proinflammatory factors and chemokines, recruit and activate immune cells including monocyte macrophages to infiltrate the infarct site, and exacerbate the inflammatory response. In the advanced stages of cardiac injury, macrophages undergo phenotypic changes and mediated tissue repair response (4, 5). Notably, an imbalance between inflammatory and reparative responses contributes to pathological cardiac remodeling (6–8).

A large number of studies have recently demonstrated that the expression of genes related to cardiac hypertrophy, fibrosis, and inflammation can be regulated by transcription, and inhibited harmful transcription and promoted beneficial transcription can effectively improve cardiac function after cardiac injury (9–11). Therefore, it is meaningful to explore the transcriptional regulatory mechanisms of macrophages in adverse cardiac remodeling after AMI.

## 2. Origin and classification of cardiac macrophages

### 2.1. Cardiac resident macrophages

In recent years, scientists have widely recognized that tissues establish a certain number of macrophages during embryonic development, called resident macrophages, which has revolutionized previous clinical beliefs of circulating monocytes entering the tissue and differentiating into macrophages (12–15). Therefore, the heart contains subpopulations of macrophages from different origins.

It has been reported that cardiac resident macrophages in mice account for approximately 7%–8% of non-cardiomyocytes in the stable state. Cardiac resident macrophages can be divided into C-C motif receptor-2 (CCR2)^−^ and CCR2^+^ macrophage subset according to CCR2. CCR2^−^ resident macrophages derived from embryonic yolk sac and fetal liver hematopoietic stem cells maintain their numbers by local proliferation. A subset of CCR2^+^ resident macrophages is supplemented by monocyte recruitment and proliferation (13, 16). In addition, based on the expression of major histocompatibility complex II (MHCII) and CCR2, there are three classifications of cardiac resident macrophages, namely, MHCII^low^ CCR2^−^, MHCII^high^ CCR2^−^, and MHCII^high^ CCR2^+^ (17).

### 2.2. Cardiac-recruited macrophages

Monocytes play an important role in cardiac damage. Monocytes can be divided into classical monocyte lymphocyte antigen 6C (Ly6C)^high^ and non-classical monocyte Ly6C^low^ in mice according to the Ly6C expression. Furthermore, mice monocytes have also been classified into classical monocytes Ly6C^high^ CCR2^high^ CX3C chemokine receptor 1 (CX3CR1)^low^ and non-classical monocytes Ly6C^low^ CCR2^high^CX3CR1^high^ (18). *In vivo* microscopy, Ly6C^high^ monocytes have been observed to circulate rapidly and recruit to the site of injury, while Ly6C^low^ monocytes circulate more slowly and crawl along the endothelium to maintain homeostasis (19).

The number of macrophages at the infarct site increases significantly after AMI, and their infiltration into the infarct site occurs in two consecutive phases. In the early stage of AMI, Ly6C^high^ monocytes are recruited to the infarct site under the action of chemokine CCL2 and differentiated into recruited CCR2^+^ macrophages, resulting in inflammatory response. Subsequently, Ly6C^high^ macrophages differentiate into Ly6C^low^ macrophages with the assistance of the orphan nuclear receptor 4a (Nr4a) and coordinated tissue repair response (20, 21).

## 3. Function of cardiac macrophages

### 3.1. Function of cardiac resident CCR2^−^ macrophages

#### 3.1.1. Facilitating cardiac electrical conduction

Resident macrophages play a significant role in cardiac electrical conduction. Hulsmans et al. proved that there are abundant resident macrophages in the atrioventricular (AV) node that are connected to cardiomyocytes *via* Cx43-containing gap junctions, and they promote cardiac electrical conduction (22). Another study reported that Amphiregulin (AREG) produced by cardiac macrophages leads to a phosphorylation of Cx43 *via* the EGFR/MEK/ERK pathway, mediating the normal formation of gap junctions, thus promoting cardiac electrical conduction (23).

#### 3.1.2. Supporting mitochondrial homeostasis

Cardiomyocytes contain a large number of mitochondria to provide energy for the heart. However, the continuous supply of energy induces the accumulation of defective mitochondria. Timely removal of detrimental mitochondria is highly important to maintain cardiac homeostasis. Melentijevic et al. discovered a novel garbage removal mechanism. *Caenorhabditis elegans* neurons secrete a specialized membrane vesicle, called exophers, which can package and transport dysfunctional proteins and organelles (24). Research has found that cardiomyocytes release dysfunctional mitochondria *via* the exophers, and cardiac resident macrophages recognize and phagocytic these exophers with the assistance of the receptor Mertk, thus preventing inflammasome activation and autophagy block, eventually maintaining cardiac homeostasis (25).

#### 3.1.3. Promoting tissue repair

Research revealed that the population of macrophages expanded significantly in response to cardiac injury and neonatal mice increased the number of CCR2^−^ resident macrophages. However, in adult mice, there was a dramatic loss of CCR2^−^ resident macrophages and a large number of infiltrated circulating monocyte-derived inflammatory CCR2^+^ macrophages. Compared with adult mice, neonatal mice induced a weaker inflammatory response, accompanied by angiogenesis and cardiomyocyte proliferation. In addition, the inhibition of cardiac monocyte recruitment in adult mice reduced inflammation response and improved cardiac function (26). Another research reported that cardiac resident macrophages repressed fibrosis and promoted angiogenesis under the condition of cardiac pressure overload (27). These results suggest that resident macrophages derived from embryonic development are key mediators in promoting cardiac repair.

#### 3.1.4. Mediating adaptive myocardial remodeling

Research has revealed that cardiac resident CCR2^−^ macrophages mediate adaptive cardiac remodeling. To be specific, exhaustion of CCR2^−^ macrophages mice impaired adaptive changes to maintain cardiac output after dilated cardiomyopathy surgery, such as depressed coronary angiogenesis and aggravated ventricular remodeling, suggesting that CCR2^−^ resident macrophages are a key protective factor. Further studies have found that CCR2^−^ macrophages interact with adjacent cardiomyocytes through the adhesion complex and sense mechanical stretch, contributing to the expression of the pro-angiogenic growth factor and promoting coronary angiogenesis depending on TRPV4. Therefore, CCR2^−^ resident macrophages may be a key mediator in regulating adaptive cardiac remodeling (28).

### 3.2. Function of cardiac resident CCR2^+^ macrophages

Cardiac resident CCR2^+^ macrophages promote the recruitment of circulating monocytes through the pathway of myeloid differentiation primary response 88 (MyD88). Resident CCR2^+^ macrophages are inflammatory macrophages but that express lower levels of inflammatory cytokines and chemokines than recruited CCR2^+^ macrophages. In addition, resident CCR2^+^ macrophages express different type I IFN-stimulated genes in response to myocardial injury compared with recruited CCR2^+^ macrophages, suggesting that CCR2^+^ macrophages respond to type I interferon during myocardial infarction. Notably, the research also found that the inhibition of resident CCR2^+^ macrophage activity can limit inflammatory response and pathological cardiac remodeling, which may be a therapeutic target to improve outcomes following AMI (29). However, little is known about the function of the resident CCR2^+^ macrophage subset, and further studies are needed to elucidate the role of cardiac resident CCR2^+^ macrophages.

### 3.3. Function of cardiac-recruited macrophages

**Figure 1:**
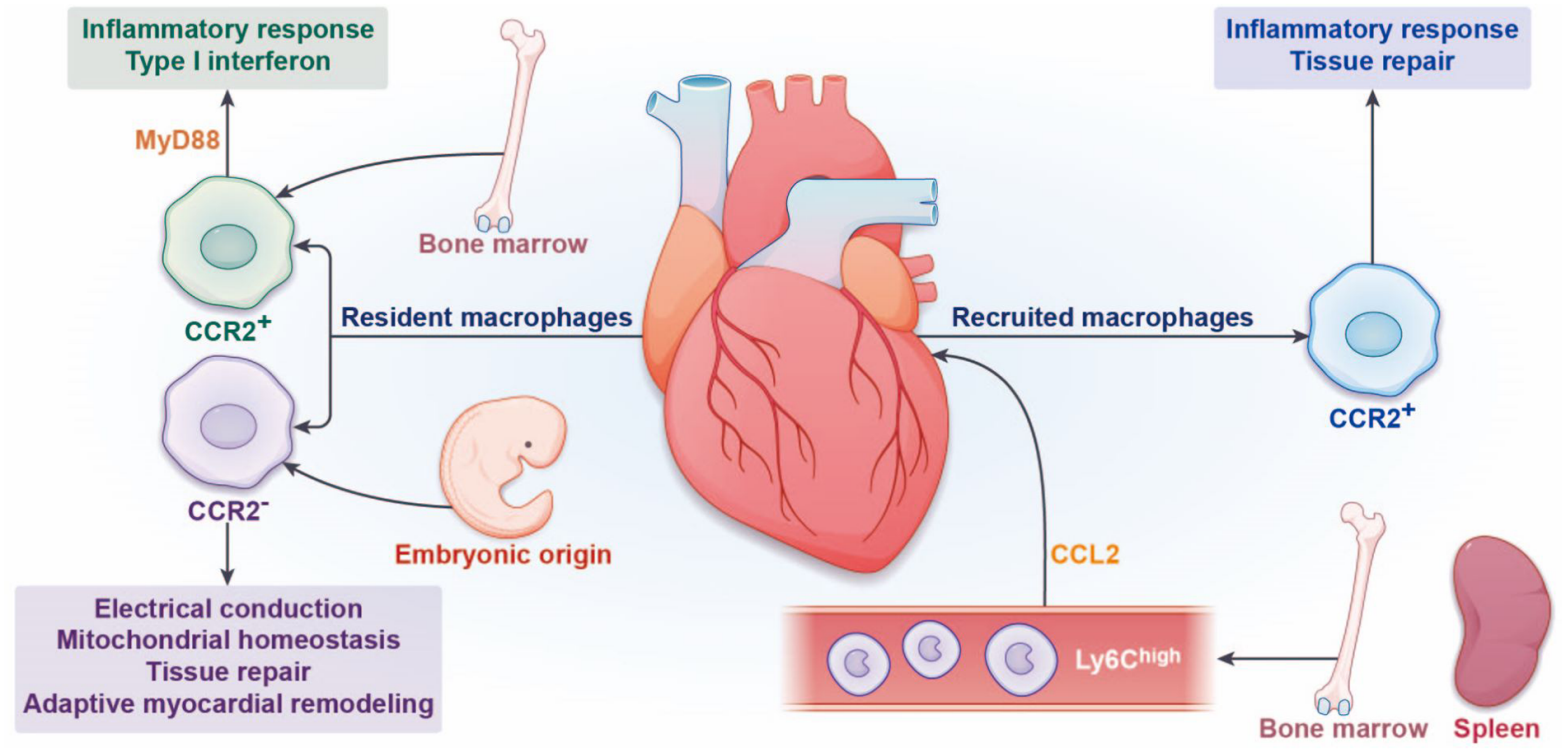
Origin and function of cardiac macrophages. In the steady state, the heart contains a certain number of resident macrophages. According to the expression of C-C motif receptor-2 (CCR2), cardiac resident macrophages can be divided into CCR2^−^ and CCR2^+^ macrophages. CCR2^−^ resident macrophages are derived from embryonic yolk sac and fetal liver hematopoietic stem cells, which maintain their numbers by local proliferation. CCR2^+^ resident macrophages promote the recruitment of circulating monocytes mainly through the pathway of MyD88. The functions of CCR2^−^ resident macrophages are facilitated cardiac electrical conduction, mitochondrial homeostasis, tissue repair, and adaptive myocardial remodeling. The functions of resident CCR2^+^ macrophages are proinflammatory response and type I interferon. During myocardial infarction, Ly6C^high^ monocytes derived from the bone marrow and spleen are recruited to the infarct site under the action of chemokine CCL2, differentiate into recruited CCR2^+^ macrophages, and mediate inflammatory response. Subsequently, repairing Ly6C^low^ macrophages mediate repair response.

Recruited macrophages show functional heterogeneity during AMI. In the early stages after AMI, Ly6C^high^ macrophages produce and release a large number of inflammatory cytokines and chemokines such as interleukin IL-1β (IL-1β), IL-6, IL-12, tumor necrosis factor-α (TNF-α), and CXC motif chemokine ligand 9 (CXCL9). All of these factors work together to promote inflammation response. In the advanced stages after AMI, Ly6C^high^ macrophages differentiate into Ly6C^low^ macrophages, mainly secrete anti-inflammatory cytokines and chemokines such as IL-10 and C-C motif chemokine ligand 17 (CCL17), and also produce vascular endothelial growth factor (VEGF) and tumor growth factor β (TGFβ) (30, 31). These mediators jointly promote the production of the extracellular matrix, cell proliferation, and angiogenesis, thereby promoting tissue repair (Figure 1).

It has also been described that recruited macrophages in the inflammatory phase are M1 macrophages, and macrophages in the repair phase are described as M2 macrophages. However, some researchers believe that the transcriptional heterogeneity of recruited macrophages does not easily conform to the M1/M2 paradigm, and the M1/M2 classification is more suitable for studies of bone marrow-derived macrophages *in vitro* (6).

## 4. Transcriptional regulation of macrophage phenotype and function in heart failure

### 4.1. Transcriptional regulation

Transcription refers to a process in which *RNA* polymerase (Pol II) and promoters or enhancers on *DNA* sequences are combined with the assistance of transcription factors (TFs), subsequently regulating gene expression (32). Research studies have demonstrated that the degree of chromatin opening is an essential factor in the regulation of transcription. Nucleosome, the basic structure of chromatin, is composed of the 147 bp segment of *DNA* and histone octamer formed by two molecules each of *H2A, H2B, H3*, and *H4*. Such a compacted nucleosome structure leads to regulatory elements such as transcription factors that cannot bind to *DNA*-induced repression of gene transcription (33, 34). Thus, the first step in gene transcription is to transform dense heterochromatin into open euchromatin. Histone modification and DNA methylation in epigenetics regulate the structure and function of chromatin and recruit transcriptional activators or inhibitors to regulate transcription.

At present, the most studied histone modifications are histone methylation and acetylation. Histone methylation means that histone methyltransferase adds one, two, or three methyl groups to histone lysine or arginine residues, and demethylase regulates the process of histone demethylation (35, 36). Histone methylation is related to transcriptional inhibition as well as transcriptional activation. For example, H3K4me2/3 (37), H3K36me1/3 (38), and H3K79me1/2 (39) are associated with transcriptional activation, and H3K27me2/3 (40) and H3K9me2/3 (41) are associated with transcriptional inhibition (42). Histone acetylation is regulated by histone acetyltransferase (HAT) and histone deacetylase (HDAC) mediates the process of deacetylation. It is well known that histone acetylation is associated with transcription activation, and histone deacetylation leads to transcriptional inhibition (43, 44). *DNA* methylation refers to binding a methyl group on the C5 position of CpG dinucleotide cytosine under the action of *DNA* methyltransferase (DNMT) and is modified into 5-methyl cytosine (5-mc). DNA demethylation is catalyzed by the enzyme Ten-Eleven translocation protein 1 (Tet1) to convert 5-mc into 5-hydroxymethylcytosine (5hmc). Most gene promoters are located on the CpG island and when the promoter at the CpG island is at a low *DNA* methylation level, resulting in gene transcription, Conversely, at high levels of DNA methylation, which inhibits promoter combined with TFs-induced gene expression silenced (45, 46) (Table 1).

**Table 1:** Histone modification and DNA methylation in epigenetics associated with transcription.

Epigenetics	Major function	Key enzyme	References
H3K4me2/3	Active transcription	KMT2, SMYD, SET7/9	(37)
H3K36me1/3	Active transcription	NSD3, SETD2	(38)
H3K79me1/2	Active transcription	DOT1	(39)
H3K27me3	Repressed transcription	PRC2, EZH2	(40)
H3K9me2/3	Repressed transcription	SUV39H1, SUV39H2, G9a, SETDB1, SETDB2, GLP	(41)
Histone acetylation	Active transcription	P300/CBP	(43, 44)
Histone deacetylation	Repressed transcription	HDAC I: HDAC1-3, 8, HDAC IIa: HDAC4, 5, 7, 9, HDAC IIb: HDAC6, HDAC10, HDAC III: SIRT1-7, HDAC IV: HDAC11	(43, 44)
DNA methylation	Repressed transcription	DNMT1, DNMT2, DNMT3a, DNMT3b	(45, 46)
DNA demethylation	Active transcription	Tet1	(45, 46)

Numerous studies have shown that epigenetic regulation affects the gene expression related to myocardial fibrosis, hypertrophy, and so on through transcription. G9a, H3K9 histone methyltransferase, combined with enhancer of zeste homolog 2 (EZH2) and transcription factor myocyte-specific enhancer factor 2C (MEF2C), inhibits the transcription of antihypertrophic genes, thereby promoting cardiac hypertrophy (47). Endothelial Kruppel-Like Factor 4 (KLF4) is an important protective regulatory factor against atherosclerosis. There is increased *DNA* methylation of the KLF4 promoter in the CpG island in atherosclerotic disease, inhibiting KLF4 transcription. In addition, DNMT inhibitors ameliorate atherosclerosis (48). Liu et al. showed that the forkhead box transcription factor P1 (Foxp1) regulated the transcription of transforming growth factor-β1 (TGF-β1)-mediated pathological cardiac remodeling (49). It has been reported that Runt-related transcription factor-1 (RUNX1), which is a key regulatory factor involved in pathologic cardiac remodeling, may be a potential target for the treatment of HF (50).

In summary, it is known that epigenetic and transcription factors regulate the expression of myocardial fibrosis and hypertrophy genes, thus participating in the process of pathological cardiac remodeling. Macrophages play an important role in cardiac remodeling. Therefore, targeting the transcriptional regulation of cardiac macrophages contributes to finding novel targets for HF treatment.

### 4.2. Possible transcriptional regulation in resident macrophages

After AMI, there is a decrease in the abundance of CCR2^−^ resident macrophages at the infarct site and subsequently a slow increase by self-proliferation. The deletion of CCR2^−^ resident macrophages using the CX3CR1-based system significantly deteriorates cardiac function and exacerbates adverse cardiac remodeling (28). There are also studies that have found that CCR2^−^ resident macrophages mediate the cardiac repair response by promoting cardiomyocyte proliferation and angiogenesis (27). In conclusion, CCR2^−^ resident macrophages play a protective role in cardiac injury. CCR2^+^ resident macrophages mainly promote the recruitment of monocytes after cardiac injury, but its role in HF is poorly studied.

There are few studies on the transcriptional regulation of cardiac resident macrophages in HF. The crucial role of resident macrophages in cardiac injury may become a hotspot in future research. It has been reported that the reduction of Legumain (Lgmn) in CCR2^−^ macrophages leads to significantly aggravated adverse cardiac remodeling post-AMI. Mechanistically, the decrease in Lgmn leads to a downregulation of the degradation and clearance of apoptotic cardiomyocytes. In addition, Lgmn deficiency also upregulates the infiltration of inflammatory macrophages, resulting in excessive inflammatory response. Conversely, Lgmn activation significantly improves cardiac function (17), suggesting that Lgmn in CCR2^−^ macrophages is a cardioprotective factor. Several studies have revealed that transcription factors such as CCAAT-enhancer-binding protein (C/EBpβ), p53, and ELK1 bind to the promoter of Lgmn and regulate the transcription of Lgmn (51, 52). Accordingly, more in-depth studies are needed to explore the transcription regulation of Lgmn in cardiac resident macrophages, so as to identify novel targets for improving cardiac function. CCR2^−^ resident macrophages mediate cardiac repair by promoting cardiomyocyte proliferation and angiogenesis. A study has shown that NLR family CARD domain containing 5 (NLRC5) and activator of transcription 3 (STAT3) promote the transcription of vascular enhancing angiopoietin-2 (Ang2) and cyclin D1 (CCND1), thereby promoting angiogenesis (53). Transcription factor GATA1 and histone methyltransferase SET7 promote the transcription of vascular endothelial growth factor–induced angiogenesis (54). In the future, targeting CCR2^−^ resident macrophages to promote cardiomyocyte proliferation and angiogenesis through transcriptional regulation may become a research hotspot.

### 4.3. Regulation of recruited macrophage phenotype and function through transcription

Persistent inflammatory response interferes with the timely occurrence of the repair response, resulting in pathological cardiac remodeling. Therefore, achieving a balance between the two phases may provide a breakthrough in the treatment of HF.

Lgr4 is a member of the leucine-rich repeat-containing G protein-coupled receptor (LGR) family that is involved in the AMP/protein kinase A (PKA)/cAMP response element binding protein (CREB) signal pathway (55, 56). Activator protein-1 (AP-1) is a proinflammatory transcription factor complex composed of Fos and Jun family members, regulating proinflammatory factors and chemokine expression to modulate macrophage polarization (57–59). Huang et al. found that macrophages highly expressed Lgr4 after AMI. Lgr4 promoted the transcription of Fos and Jun families through the cAMP/PKA/CREB pathway, synergistically increased the activity of AP-1, and promoted the downstream proinflammatory gene transcription, thus aggravating cardiac inflammatory response and poor cardiac remodeling. However, a low expression of Lgr4 plays a cardioprotective role through augmenting reparative macrophage subsets (60). Yes-associated protein (YAP) and transcriptional coactivator with PDZ-binding motif (TAZ) are key regulators of hippo signaling pathways, involved in cardiac regeneration and repair (61, 62). Mia et al. reported that YAP/TAZ bound to the promoter of proinflammatory factors *IL-6* and facilitated *IL-6* transcription, whereas YAP/TAZ inhibited reparative gene Arginase-1 (*Arg1*) transcription. This occurs because YAP/TAZ recruits histone deacetylase 3 (HDAC3) and nuclear receptor corepressor 1 (NCoR1) to form the transcriptional repressor complex and then binds to the *Arg1* promoter. More importantly, the reduction of YAP/TAZ leads to a polarization of macrophages toward the reparative phenotype, which ameliorates cardiac fibrosis and hypertrophy and enhances angiogenesis after AMI (63). Histone lactylation, a newly discovered epigenetic regulation, is a process by which lactyl groups are added to histone lysine residues to modulate the chromatin structure and facilitate gene transcription (64). Wang et al. reported that an increase in histone H3K18 lactylation (H3K18la) levels in infiltrating macrophages post-AMI and the repair genes *Lrg1, Vegf-a*, and *IL-10* are the downstream targets of H3K18la. Treatment with sodium lactate upregulates H3K18la levels, enhances *Lrg1*, *Vegf-a*, and *IL-10* expression, inhibits inflammation, improves angiogenesis, and restrains cardiac fibrosis and pathological remodeling after AMI. This research also found that lactate transporter monocarboxylate transporter 1 (MCT1) provides abundant substrates for histone lactylation by transporting extracellular lactates into the cell. In order to further elucidate the upstream regulatory mechanism of H3K181a, it was found that histone acetyltransferase GCN5 could catalyze the process of H3K18la and promote repair gene *Lrg1, Vegf-a,* and *IL-10* transcription, thereby improving cardiac function *via* anti-inflammatory response and promoting angiogenesis (65). The above findings suggest that H3K181a has cardioprotective effects by promoting the transcription of reparative genes. The timely resolution of inflammation and initiation of cardiac repair after AMI are crucial for heart repair. Cardioprotective transcription factors and epigenetic regulators can promote repair gene transcription, subsequently affecting macrophage phenotype and function. These transcription factors and epigenetic regulators may be potential therapeutic targets for treating HF.

**Figure 2:**
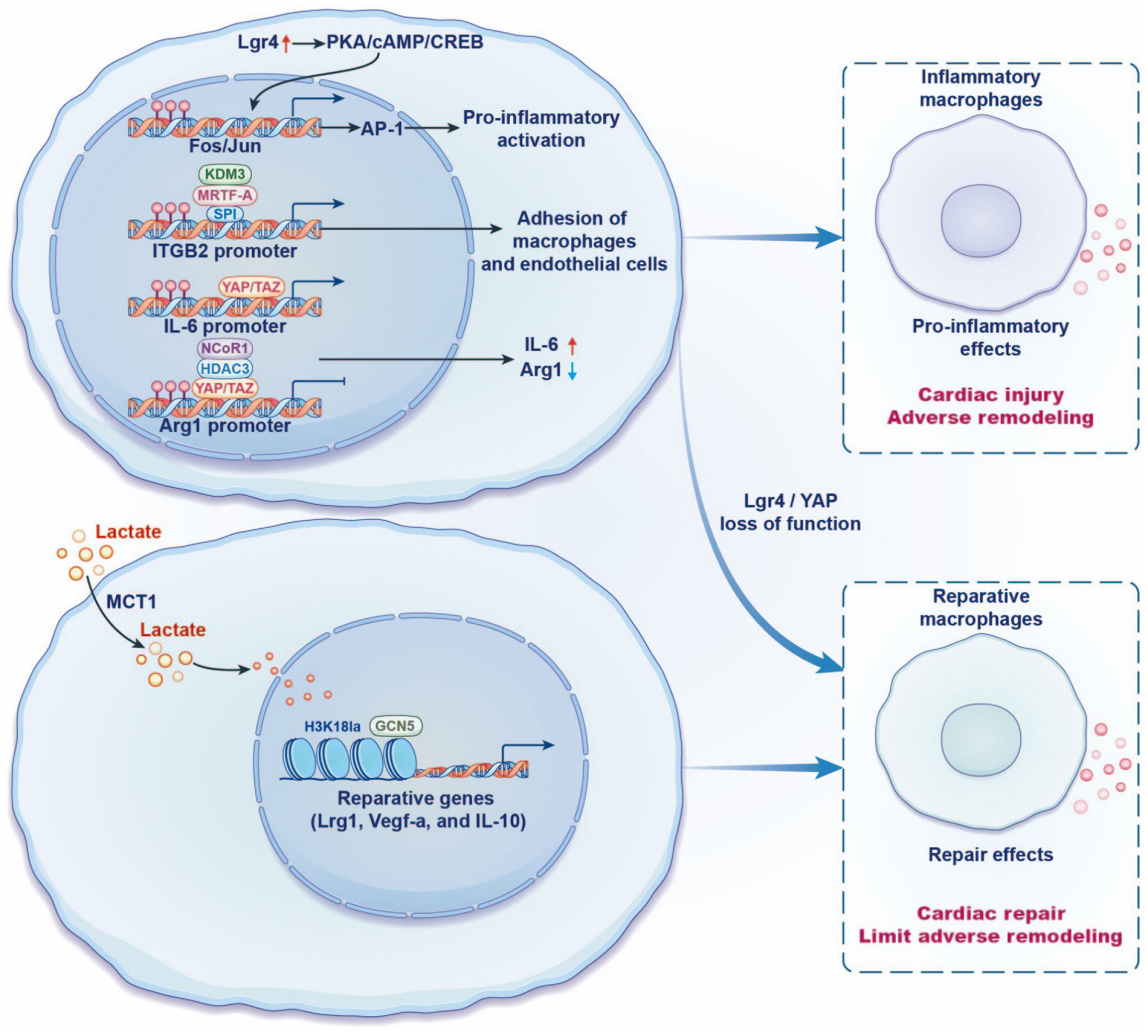
Regulation of recruited macrophage phenotypes and function in heart failure by transcription. Lgr4 promotes the transcription of Fos and Jun families through the cAMP/PKA/CREB pathway, synergistically increases the activity of AP-1, and promotes the downstream proinflammatory gene expression, thus aggravating cardiac inflammatory response and poor cardiac remodeling. YAP/TAZ binds to the proinflammatory cytokine IL-6 promoter, whereas YAP/TAZ inhibits Arg1 expression by binding to the Arg1 promoter and recruiting the histone deacetylase 3 (HDAC3)-nuclear receptor corepressor 1 (NCoR1) inhibition complex. Lgr4/YAP loss of function has cardioprotective effects. MRTF-A in macrophages collaborates with transcription factor Sp1 and histone H3K9 demethylase KDM3A to promote integrin β2 (ITGB2) transcription, leading to an increase in the adhesion of macrophages to endothelial cells, eventually resulting in inflammatory response. Lactate transporter monocarboxylate transporter 1 (MCT1) provides abundant substrates for histone H3K18 lactylation (H3K18la) and promotes the repair genes *Lrg1*, *Vegf-a*, and *IL-10* transcriptional. It is further found that histone acetyltransferase GCN5 is able to catalyze the process of histone lactylation, thereby improving cardiac function *via* an anti-inflammatory response.

Macrophages interact with adhesion molecules on the surface of endothelial cells through their own specific receptors (e.g., integrins), thereby gaining access to the vasculature and initiating the proinflammatory response (66, 67). Therefore, blocking the adhesion of macrophages to endothelial cells may attenuate the inflammatory response. Liu et al. found that MRTF-A in macrophages collaborates with transcription factor Sp1 and histone H3K9 demethylase KDM3A to promote integrin β2 (ITGB2) transcription, leading to an increase in the adhesion of macrophages to endothelial cells, eventually enhancing inflammatory response and cardiac hypertrophy following cardiac injury. However, MRTF-A deficiency in macrophages reverses the above transcription process, resulting in alleviating inflammatory response and cardiac hypertrophy (68), thereby indicating that suppression of the interaction between macrophages and endothelial cells to attenuate the inflammatory response is a novel therapeutic idea for HF (Figure 2). In addition to the above transcription processes, several widely studied transcription factors have been shown to participate in the process of macrophage phenotype transition, such as nuclear factor-κB (NF-κB), signal transducer and activator of transcription 6 (STAT6), and so on. In the case of pathological stress, the IKK complex is activated, further phosphorylating the IκB protein, ultimately leading to NF-κB entering the nucleus and binding to specific DNA sequence-induced proinflammatory macrophage activation (69, 70). IL-4-activated STAT6 acts as a transcriptional repressor in macrophages, limiting IL-1β transcription and inflammasome activation, resulting in a weaker inflammatory response (71). Therefore, the research and development of novel drugs targeting these transcription factors may be promising for the treatment of HF.

Macrophage metabolism is another important pathway that regulates macrophage phenotype and function through transcriptional pathways. Metabolic reprogramming was first proposed by Otto Warburg, who discovered that tumor cells rapidly produce ATP through the glycolytic pathway even under aerobic conditions, accompanied by a decrease in oxidative phosphorylation (72). This similar process was also found in macrophages. When macrophages are stimulated by lipopolysaccharide (LPS), metabolic pathways are characterized by enhanced glycolysis and a broken tricarboxylic acid (TCA) cycle, and macrophages present a proinflammatory phenotype. When macrophages are stimulated by IL-4, metabolic pathways are characterized by enhanced fatty acid oxidation and oxidative phosphorylation, and macrophages exhibit an anti-inflammatory phenotype (73). Research has demonstrated that α-ketoglutarate (αKG) generated from glutamine is an anti-inflammatory mediator. Specifically, αKG promotes the expression of repair genes Arg1, Yml, Retnla, and Mrcl through JMJD3-dependent H3K27 demethylation epigenetic pathways, resulting in promoting repair macrophage activation. Second, glutamine metabolism regulates IKKβ activity through αKG-PHD, in turn, suppressing the NF-κB pathway, thereby inhibiting inflammatory macrophage activation. In summary, αKG is an important metabolite that regulates macrophage polarization through metabolic and epigenetic mechanisms (74). BMDM stimulated by LPS leads to the accumulation of succinate, an intermediate metabolite in the TCA cycle, which stabilizes hypoxia-inducible factor-1α (HIF-1α) and subsequently promotes the transcription of IL-1β, inducing inflammatory response (75). DeBerge et al. found that HIF-1α mediates poor cardiac remodeling after AMI. In terms of mechanism, HIF-1α promotes myeloid-epithelial-reproductive receptor tyrosine kinase (MerTK) cleavage through macrophage glycolytic reprogramming. MerTK is a cardioprotective receptor that mediates the clearance of apoptotic cardiomyocytes to promote macrophage reparative phenotype activation. Conversely, a knockdown of HIF-1α in bone marrow cells antagonizes the deterioration of cardiac function after AMI (76). The compound dimethyl fumarate (DMF) significantly improves cardiac function post-AMI, which leads to a decrease in the expression of HIF-1α and IL-1β, but is also accompanied by an increase in macrophage oxidative phosphorylation levels (77). The above studies suggest that macrophage metabolic reprogramming is one of the mechanisms regulating macrophage phenotype and function. More importantly, the transcription factors HIF-1α and NF-κB and the epigenetic regulators H3K27 are involved in the process of metabolic reprogramming.

## 5. Treatment of HF with traditional Chinese medicine through transcriptional regulation

Traditional Chinese medicine (TCM) has the advantages of multiple targets, few side effects, and remarkable therapeutic effects. More importantly, it has been used in clinical practice for thousands of years. The molecular nature of TCM has not yet been fully understood, but it is usually a mixture of multiple compounds that work together to exert therapeutic effects. The sources of TCM are mainly plants, animals, and minerals, which undergo specific processing and preparation processes to enhance therapeutic properties and reduce toxicity. They are usually given to patients in the form of decoction, powder, capsule, or tablet. Although clinical studies have proven the effectiveness of TCM in treating diseases, the specific pathways and modes of action of these drugs are still being studied.

Several studies have found that the treatment of HF with TCM is done through transcriptional regulation. Nuanxinkang (NXK) is composed of Red Ginseng (dry root and rhizome of *Panax ginseng* C.A. Mey) and Holly (root of *Ilex pubescens* Hook. et Arn). Dong et al. found that NXK improves cardiac function by inhibiting the NF-κB signaling pathway to suppress the proinflammatory macrophage phenotype and promote anti-inflammatory macrophage phenotype (78). Ankyrin repeat domain 1 (ANKRD1), a nuclear transcription co-factor, induces the transcription of hypertrophic genes and is involved in pathological cardiac remodeling (79). Baoyuan decoction (BYD) consists of *Astragalus membranaceus* (Fisch.) Bunge, *Panax ginseng* C. A. Mey., *Glycyrrhiza uralensis* Fisch., and *Cinnamomum cassia* Presl. Baoyuan decoction (BYD) inhibits the transcription of myocardial fibrosis and hypertrophy genes through ANKRD1 (80). Curcumin, a polyphenol, originates from the plant curcuma longa, which ameliorates heart hypertrophy and inhibits the development of heart failure by inhibiting p300-HAT activity (81). Nuclear factor erythroid 2-related factor 2 (Nrf2) is a key transcription factor in antioxidant stress response. Tanshinone I (Tan I) is an active ingredient extracted from the Chinese herb Danshen, and it has been shown to upregulate the phosphorylation of AKT and promote the Nrf2 shuttle into the nucleus, activate the expression of its downstream antioxidant enzymes, and then relieve oxidative stress and mitochondrial damage to improve cardiac function (82). On the one hand, the above studies indicate that TCM has a remarkable and beneficial effect in AMI treatment, and on the other hand, transcription factors and epigenetic regulatory factors can be used as key targets to treat diseases. In addition to TCM treatment, biologics and nanoparticles that deliver therapeutic substances and gene editing to macrophages are rapidly evolving, which will contribute to the treatment of heart failure more effectively in the future (3).

Although TCM is not a traditional treatment for cardiovascular disease in Western medicine, a growing number of studies suggest that TCM has remarkable therapeutic effects in lowering blood pressure, improving cholesterol levels, and reducing inflammation (83–85). Therefore, more research is needed to fully understand the mechanisms and potential role of TCM in the treatment of cardiovascular disease, which will contribute to the widespread use of TCM.

## 6. Summary

As mentioned above, the targeted regulation of macrophage phenotype by repressing inflammatory gene transcription or promoting repair gene transcription will lead to an amelioration of adverse cardiac remodeling. Accordingly, research and development novel drugs targeting cardioprotective transcription factors and epigenetic regulators factors may hold promise for HF treatment. At present, there are few studies on cardiac resident macrophages. In particular, CCR2^−^ resident macrophages play protective roles in promoting cardiomyocyte proliferation, angiogenesis, cardiac conduction, and mitochondrial homeostasis. Therefore, efforts should be devoted to the transcription regulation of CCR2^−^ resident macrophages in the future.
